# Supplementary material for: Favorable Effect of High-Density Lipoprotein Cholesterol on Gastric Cancer Mortality by Sex and Treatment Modality
Source: Cancers (Basel). 2023 Apr 25;15(9):2463. doi: 10.3390/cancers15092463 (PMC10177473; doi:10.3390/cancers15092463)
Supplement: Supplementary file 1 [file cancers-15-02463-s001.zip › cancers-2260328-supplementary.pdf]

Supplementary Materials

Missing data in KNUH cohort

The missing rate of body mass index was 0.88% (30/3379) among the final eligible population. Therefore, we deleted missing data list-wise in the adjusted analysis.

The missing rate of fasting glucose was 9.3% among the final eligible population. Therefore, we put missing values as unknown group in the adjusted analysis. Thus, fasting glucose group was classified into three groups; <126, ≥126, unknown.

Supplementary Table S1. Different definitions of HDL-C categories

**HDL-C (Main definition)**	HDL-C, mg/dL
	<40
	40-49
	50-59
	60-69
	≥70
**HDL-C by ATP III**	
Low	<40 in men, <50 in women
Normal	≥40 in men, ≥50 in women
**HDL-C by NECP**	
Low	<40 in men, <50 in women
Intermediate	40-59 in men, 50-59 in women
High	≥60
**HDL-C by 4 groups**	
Very low	<30
Low	30-40 in men, 30-50 in women
Intermediate	40-59 in men, 50-59 in women
High	≥60

ATP III, Adult Treatment Panel III; HDL-C, high density lipoprotein cholesterol; NECP, National Cholesterol Education Program.

Supplementary Table S2. Women factors of Gastric Cancer Patients by HDL-C (NHISS cohort)

	**HDL-C, mg/dL**				
	**<40 (n=722)**	**40-49 (n=1920)**	**50-59 (n=2213)**	**60-69 (n=1422)**	**≥70 (n=971)**
Menopausal status					
Premenopausal	63 (8.87)	203 (10.75)	259 (11.85)	217 (15.46)	165 (17.21)
Hysterectomy	50 (7.04)	165 (8.74)	174 (7.96)	100 (7.12)	79 (8.24)
Postmenopausal	597 (84.08)	1520 (80.51)	1753 (80.19)	1087 (77.42)	715 (74.56)
Estrogen replacement therapy in post-menopausal women*					
Never	507 (85.07)	1306 (86.03)	1464 (83.66)	918 (84.53)	593 (82.94)
< 2 yr	34 (5.70)	19 (5.99)	129 (7.37)	83 (7.64)	62 (8.67)
≥2 and <5yr	12 (2.01)	34 (2.24)	53 (3.03)	32 (2.95)	17 (2.38)
≥5yr	17 (2.85)	28 (1.84)	45 (2.57)	27 (2.49)	18 (2.52)
Unknown	26 (4.36)	59 (3.89)	59 (3.37)	26 (2.39)	25 (3.50)
Delivery frequency					
1	46 (6.48)	118 (6.25)	138 (6.31)	101 (7.21)	73 (7.62)
2	651 (91.69)	1722 (91.21)	1992 (91.08)	1262 (90.08)	863 (90.08)
3	13 (1.83)	48 (2.54)	57 (2.61)	38 (2.71)	22 (2.30)
Breast feeding duration					
<6M	37 (5.25)	122 (6.55)	141 (6.55)	126 (9.10)	110 (11.53)
≥6M and <1yr	85 (12.06)	266 (14.27)	339 (15.75)	238 (17.18)	171 (17.92)
≥1yr	544 (77.16)	1375 (73.77)	1538 (71.47)	927 (66.93)	606 (63.52)
Never	39 (5.53)	101 (5.42)	134 (6.23)	94 (6.79)	67 (7.02)
Oral pill					
Never	583 (82.00)	1528 (80.80)	1807 (82.66)	1139 (81.18)	762 (79.29)
<1yr	54 (7.59)	155 (8.20)	177 (8.10)	114 (8.13)	98 (10.20)
≥1yr	41 (5.77)	119 (6.29)	120 (5.49)	90 (6.41)	52 (5.41)
Unknown	33 (4.64)	89 (4.71)	82 (3.75)	60 (4.28)	49 (5.10)

HDL-C, high density lipoprotein cholesterol.
* Missing data is premenopausal women.

Supplementary Table S3. Baseline Characteristics of Gastric Cancer Patients (NHISS cohort)

	**Alive (n= 17432)**	**Death (n= 5086)**	**p-values***
Men, no (%)	11471 (66.2)	3376 (74.2)	<0.001
Age, mean (SD)	63.09 (9.3)	69.83 (9.7)	<0.001
Economic status, mean (SD)†	12.3 (5.9)	12.1 (5.9)	0.125
HDL-C, mg/dL, no (%)			
<40	2609 (15.0)	953 (18.7)	<0.001
40-49	5359 (30.8)	1611 (31.7)	
50-59	4794 (27.5)	1339 (26.3)	
60-59	2823 (16.2)	729 (14.3)	
≥70	1796 (10.33)	455 (8.94)	
BMI, kg/m^2, mean (SD)	24.06 (3.0)	23.06 (3.1)	<0.001
BMI, kg/m^2, no (%)			
<18.5	379 (2.2)	330 (6.5)	<0.001
18.5-22.4	5916 (34.0)	2187 (43.0)	
22.5-24.9	4711 (27.1)	1247 (24.5)	
25-29.9	5853 (33.7)	1215 (23.9)	
≥30	520 (3.0)	108 (2.1)	
Hypertension, no (%)	6900 (39.7)	2223 (43.7)	<0.001
Stroke, no (%)	316 (1.8)	156 (3.1)	<0.001
Heart disease, no (%)	753 (4.3)	314 (6.2)	<0.001
DM, no (%)	2658 (15.3)	1028 (20.2)	
Smoking status, no (%)			
Never	9201 (53.0)	2578 (50.7)	0.002
Past	4412 (25.4)	1328 (26.1)	
Current	3753 (21.6)	1184 (23.3)	
Drinking frequency, no (%)			
None	9861 (56.8)	3206 (63.0)	<0.001
1/week	4035 (23.2)	890 (17.5)	
2-3/week	2004 (11.5)	439 (8.6)	
4-5/week	768 (4.4)	253 (5.0)	
≥6/week	695 (4.0)	303 (6.0)	
Family History of GC, no (%)	2350 (13.6)	483 (9.5)	<0.001
Moderate activity, no (%)‡			

None	10289 (59.3)	3522 (69.3)	<0.001
1-2 days/week	3033 (17.5)	680 (13.4)	
3-5 days/week	2836 (16.3)	551 (10.8)	
6-7 days/week	1207 (7.0)	333 (6.6)	
**Women factors**			
Estrogen replacement therapy			
Never	3773 (83.2)	1004 (89.9)	<0.001
Less than 2 yr	360 (7.9)	37 (3.3)	
2-5yr	137 (3.0)	11 (1.0)	
≥5yr	117 (2.6)	17 (1.5)	
Unknown	147 (3.2)	48 (4.3)	
Breast feeding duration			
<6M	466 (8.1)	70 (5.5)	<0.001
6M-1yr	926 (16.1)	170 (13.3)	
≥1yr	3997 (69.4)	980 (76.4)	
Never	371 (6.4)	63 (4.9)	
Oral pill			
Never	4706 (83.7)	1100 (84.6)	0.004
<1yr	547 (8.9)	81 (6.2)	
≥1yr	355 (6.1)	63 (4.8)	
Unknown	256 (4.4)	57 (4.4)	

*p-values are derived from t-test or chi-square test. †Economic status refers 20 tile of income (score 1-20). 1 means the lowest 5%, and 20 is the highest 5%. ‡Moderate activity means physical activity with light sweating over 30 minutes in a day. For example, brisk walking, jogging, swimming leisurely, bicycling with light effort, gardening, and some housework such as vacuuming.

BMI, body mass index; DM, diabetes mellitus; GC, gastric cancer; HDL-C, high-density lipoprotein cholesterol; SD, standard deviation.

Supplementary Table S4. Baseline Characteristics of Gastric Cancer Patients in included and excluded persons (KNUH cohort)

**Variables**	**Included persons (n=3379)**	**Excluded person (n= 2582)**
Male, no (%)	2253 (66.7)	1716 (66.5)
Age, yr, mean (SD)	61.4 (11.1)	60 (12.1)
BMI, kg/m^2, mean (SD)	23.6 (3.1)	22.9 (3.2)
Glucose, mean (SD)	116.2 (43.3)	109.7 (38.6)
Tumor location, no (%)		
Distal third	1458 (43.15)	1152 (44.62)
Middle third	1450 (42.91)	1010 (39.12)
Upper third	468 (13.85)	414 (16.03)
Differentiation, no (%)		
Differentiated	1932 (57.18)	1076 (41.67)
Undifferentiated	1443 (42.7)	1496 (57.94)
Stage, no (%)		
Stage I	2773 (82.07)	1507 (58.37)
Stage II	245 (7.25)	275 (10.65)
Stage III	201 (5.95)	247 (9.57)
Stage IV	154 (4.56)	536 (20.76)
Final treatment method, no (%)		
Endoscopic resection	923 (27.32)	136 (5.27)
Curative gastrectomy	2358 (69.78)	2025 (78.43)
Palliative chemotherapy	74 (2.19)	285 (11.04)
Palliative surgery	24 (0.71)	136 (5.27)

BMI, body mass index; SD, standard deviation.

Supplementary Table S5. Baseline Characteristics of Gastric Cancer Patients by HDL-C (KNUH cohort)

	HDL-C, mg/dL				
	<40 (n=1006)	40-49 (n=1001)	50-59 (n=719)	60-69 (n=359)	≥70 (n=294)
Person years	5752.0	6190.4	4579.4	2422.8	2102.0
Men, no (%)	796 (79.1)	681 (68.0)	440 (61.2)	186 (51.8)	150 (51.0)
Age, mean (SD)	63.2 (10.1)	62.0 (10.6)	60.6 (11.5)	60.1 (11.8)	56.6 (12.1)
BMI, kg/m^2, mean (SD)	24.1 (3.0)	23.8 (3.2)	23.1 (2.92)	23.1 (3.05)	22.9 (3.0)
Glucose, mean (SD)	124.9 (53.0)	117.7 (44.9)	111.6 (36.1)	106.6 (28.1)	106.6 (25.3)
HDL-C, mean (SD)	32.3 (5.8)	44.3 (2.8)	53.99 (2.87)	63.6 (2.8)	85.65 (15.1)
BMI, kg/m^2, no (%)					
<18.5	31 (3.1)	36 (3.6)	40 (5.6)	13 (3.7)	18 (6.2)
18.5-22.4	300 (30.2)	357 (35.9)	313 (43.6)	172 (49.0)	134 (45.9)
22.5-24.9	294 (29.6)	258 (25.9)	196 (27.3)	76 (21.7)	74 (25.3)
25-29.9	341 (34.3)	322 (32.4)	159 (22.1)	85 (24.2)	61 (20.9)
≥30	27 (2.7)	22 (2.2)	10 (1.4)	5 (1.4)	5 (1.7)
Diabetes mellitus	263 (29.9)	202 (22.4)	119 (17.9)	40 (11.9)	39 (13.9)
Stage					
Stage 1	821 (81.9)	837 (83.7)	576 (80.2)	293 (81.8)	246 (83.7)
Stage 2	58 (5.8)	70 (7.0)	65 (9.1)	30 (8.4)	22 (7.5)
Stage 3	57 (5.7)	63 (6.3)	45 (6.3)	21 (5.9)	15 (5.1)
Stage 4	67 (6.7)	30 (3.0)	32 (4.5)	14 (3.9)	11 (3.7)
Location					
Upper third	478 (47.6)	455 (45.5)	277 (38.6)	128 (35.7)	120 (40.8)
Lower/mid body	395 (39.3)	429 (42.9)	328 (45.7)	169 (47.1)	129 (43.9)
Antrum	132 (13.1)	116 (11.6)	113 (15.7)	62 (17.3)	45 (15.3)
Treatment					
Endoscopic resection	399 (39.7)	301 (30.1)	135 (18.8)	58 (16.2)	29 (9.9)

Curative gastrectomy	553 (55.0)	681 (68.0)	569 (79.1)	293 (81.6)	262 (89.1)
Palliative chemotherapy	42 (4.2)	16 (1.6)	9 (1.3)	6 (1.7)	2 (0.7)
Palliative surgery	12 (1.2)	3 (0.3)	6 (0.8)	2 (0.6)	1 (0.3)
Differentiation					
Differentiated	673 (66.9)	632 (63.3)	355 (49.4)	161 (45.0)	111 (37.9)
Undifferentiated	333 (33.1)	367 (36.7)	364 (50.6)	197 (55.0)	182 (62.1)

BMI, body mass index; HDL-C, high density lipoprotein cholesterol; SD, standard deviation;

Supplementary Table S6. Hazard ratio (univariate analysis): NHISS cohort

	total		men		women	
Parameter	HR (95% CI)	P-value	HR (95% CI)	P-value	HR (95% CI)	P-value
HDL-C, mg/dL						
<40	1					
40-50	0.84 (0.78-0.91)	<0.001	0.69 (0.56-0.85)	0.001	0.62 (0.40-0.97)	0.036
50-60	0.79 (0.73-0.86)	<0.001	0.62 (0.51-0.76)	<0.001	0.53 (0.35-0.82)	0.004
60-70	0.74 (0.70-0.81)	<0.001	0.63 (0.52-0.78)	<0.001	0.44 (0.29-0.68)	<0.001
>=70	0.72 (0.65-0.81)	<0.001	0.60 (0.49-0.73)	<0.001	0.41 (0.27-0.64)	<0.001
BMI kg/m^2						
<18.5	3.33 (2.95-3.76)	<0.001	3.81 (3.32-4.38)	<0.001	2.31 (1.79-2.99)	<0.001
18.5-22.4	1.67 (1.56-1.79)	<0.001	1.82 (1.68-1.98)	<0.001	1.35 (1.18-1.54)	<0.001
22.5-24.9	1.24 (1.15-1.34)	<0.001	1.32 (1.20-1.44)	<0.001	1.07 (0.91-1.24)	0.423
25-29.9	1		1			
≥30	1.00 (0.82-1.22)	0.986	1.04 (0.80-1.34)	0.785	1.00 (0.74-1.37)	0.985
Women	0.72 (0.67-0.76)	<0.001				
age	1.08 (1.07-1.08)	<0.001	1.08 (1.08-1.09)	<0.001	1.06 (1.05-1.07)	<0.001
Income	1 (0.99-1.00)	0.148	0.99 (0.99-1.00)	0.022	1 (0.99-1.01)	0.812
Hypertension	1.15 (1.09-1.21)	<0.001	1.09 (1.02-1.16)	0.01	1.32 (1.18-1.47)	<0.001
Heart disease	1.38 (1.23-1.54)	<0.001	1.23 (1.07-1.41)	0.003	1.79 (1.46-2.21)	<0.001
Diabetes	1.34 (1.25-1.43)	<0.001	1.29 (1.19-1.39)	<0.001	1.42 (1.23-1.63)	<0.001
Stroke	1.55 (1.33-1.82)	<0.001	1.57 (1.32-1.87)	<0.001	1.37 (0.95-1.98)	0.089
Moderate activity						
None	1		1		1	
1-2 days/week	0.69 (0.64-0.75)	<0.001	0.66 (0.6-0.73)	<0.001	0.70 (0.59-0.83)	<0.001
3-5 days/week	0.60 (0.55-0.66)	<0.001	0.59 (0.53-0.66)	<0.001	0.58 (0.48-0.70)	<0.001
6-7 days/week	0.83 (0.74-0.92)	0.001	0.85 (0.75-0.96)	0.008	0.55 (0.41-0.75)	<0.001
Smoking status						
Never	1		1		1	
Past	1.06 (0.99-1.13)	0.115	0.83 (0.77-0.9)	<0.001	0.91 (0.55-1.5)	0.71
Current	1.1 (1.03-1.18)	0.007	0.87 (0.81-0.94)	0.001	0.96 (0.66-1.39)	0.821
Drinking frequency, no (%)						
None	1		1		1	
1/week	0.71 (0.66-0.76)	<0.001	0.6 (0.55-0.65)	<0.001	0.68 (0.55-0.85)	0.001
2-3/week	0.70 (0.63-0.77)	<0.001	0.59 (0.53-0.65)	<0.001	0.39 (0.21-0.71)	0.002
4-5/week	1.00 (0.88-1.14)	0.954	0.81 (0.71-0.93)	0.002	1.09 (0.48-2.47)	0.844

≥6/week	1.27 (1.13-1.43)	<0.001	1.04 (0.92-1.18)	0.51	0.40 (0.10-1.54)	0.183
Family History of GC	0.70 (0.63-0.76)	<0.001	0.7 (0.63-0.78)	<0.001	0.71 (0.60-0.85)	<0.001
Lipid lowering drug	0.70 (0.59-0.82)	<0.001	0.8 (0.66-0.98)	0.029	0.62 (0.46-0.83)	0.001
Women factors						
Menopausal status						
Pre-menopause					1	
Hysterectomy					0.93 (0.68-1.25)	0.61
Post-menopause					1.62 (1.33-1.96)	<0.001
Estrogen replacement therapy						
Never					1	
Less than 2 yr					0.41 (0.30-0.57)	<0.001
2-5yr					0.33 (0.18-0.60)	<0.001
≥5yr					0.56 (0.35-0.90)	0.016
Unknown					1.19 (0.89-1.58)	0.245
Breast feeding duration						
<6M					1	
6M-1yr					1.20 (0.91-1.59)	0.191
≥1yr					1.57 (1.23-2.00)	<0.001
Never					1.13 (0.8-1.59)	0.484
Oral pill						
Never					1	
<1yr					0.69 (0.55-0.87)	0.001
≥1yr					0.78 (0.60-1.00)	0.053
Unknown					0.95 (0.73-1.24)	0.715

BMI, body mass index; CI, confidence interval; GC, gastric cancer; HDL-C, high density lipoprotein cholesterol; HR, hazard ratio.

Supplementary Table S7. Subgroup analysis in stage I

	Unadjusted analysis			Adjusted analysis					
	Total			Total (n=2773)		Men (n=1836)		Women (n=937)	
	**Event / total No**	**HR (95% CI)**	***P***	**aHR (95% CI)**	**P^{*}**	**aHR (95% CI)**	**$P^{\dagger}$**	**aHR (95% CI)**	**$P^{\dagger}$**
HDL-C, mg/dL									
<40	129/821	1		1		1		1	
40-49	97/837	0.67 (0.51, 0.87)	0.003	0.7 (0.53, 0.92)	0.011	0.69 (0.51, 0.94)	0.019	0.7 (0.38, 1.28)	0.243
50-59	57/576	0.55 (0.41, 0.76)	<0.001	0.62 (0.45, 0.86)	0.004	0.73 (0.52, 1.04)	0.082	0.23 (0.09, 0.55)	0.001
60-69	22/293	0.39 (0.25, 0.62)	<0.001	0.48 (0.3, 0.76)	0.002	0.52 (0.3, 0.9)	0.021	0.33 (0.14, 0.81)	0.015
≥70	14/246	0.28 (0.16, 0.48)	<0.001	0.35 (0.19, 0.64)	<0.001	0.43 (0.21, 0.89)	0.029	0.19 (0.06, 0.58)	0.003
BMI, kg/m^2									
<18.5	29/89	4.34 (2.83, 6.67)	<0.001	4.02 (2.59, 6.24)	<0.001	4.6 (2.8, 7.56)	<0.001	3.04 (1.11, 8.35)	0.031
18.5-22.9	120/1020	1.39 (1.04, 1.85)	0.026	1.25 (0.93, 1.68)	0.137	1.36 (0.96, 1.92)	0.084	0.86 (0.48, 1.56)	0.625
23-24.9	81/749	1.21 (0.89, 1.66)	0.229	1.17 (0.86, 1.61)	0.318	1.41 (0.98, 2.02)	0.065	0.57 (0.28, 1.17)	0.127
25-29.9	75/829	1		1		1		1	1
≥30	9/60	1.96 (0.98, 3.91)	0.057	2.15 (1.07, 4.33)	0.031	2.75 (1.24, 6.1)	0.013	1.22 (0.28, 5.34)	0.791

*Adjusted for age, sex, body mass index, HDL, diabetes, tumor location, differentiation, and treatment method.

†Adjusted for age, body mass index, HDL, diabetes, tumor location, differentiation, and treatment method.

aHR, adjusted hazard ratio; BMI, body mass index; CI, confidence interval; HDL-C, high density lipoprotein cholesterol; HR, hazard ratio.

Supplementary Table S8. Gastric cancer death by treatment; Subgroup analysis in stage I

	Gastrectomy (n=1853)			ESD (n=919)		
	**HR (95% CI)** *	**p-value**	**P for trend**	**HR (95% CI)** *	**p-value**	**P for trend**
**HDL-C by ATP III**						
**Low**	1			1		
**Normal**	0.56 (0.43, 0.75)	<0.001		0.58 (0.37, 0.89)	0.0127	
**HDL-C by 4 groups**						
**Very low (<30)**	1			1		
**Low**	0.72 (0.45, 1.14)	0.160		0.62 (0.32, 1.19)	0.152	
**Intermediate**	0.47 (0.3, 0.72)	0.001		0.48 (0.24, 0.94)	0.032	
**High (≥60)**	0.39 (0.23, 0.66)	0.001	<0.001	0.05 (0.01, 0.42)	0.005	0.028

* Adjusted for age, sex, BMI, fasting glucose, stage, location, and differentiation. aHR, adjusted hazard ratio; BMI, body mass index; CI, confidence interval; ESD, endoscopic submucosal dissection; HDL-C, high density lipoprotein cholesterol. Definition of HDL-C was provided in Supplementary Table S1.

Supplementary Table S9. Summary of previous studies for the association between HDL-C and gastric cancer death

Author year	Reference number	Location	Design	Sample size (FU)	Results
Tamura 2012	9	Japan	Cohort	184 gastrectomy (at least 5 years after surgery or until patient death)	Death: aHR 3.61 for low HDL-C group comparing to normal HDL-C
Shen 2020	17	China	Cohort	258 gastrectomy (unclear)	Low level of HDL-C in gastric cancer correlates with cancer progression but not survival. HDL-C (<54.2 vs. ≥54.2) aHR 1.006 (0.978-1.034)
Li 2022	16	China	Cohort	431 gastrectomy (mean, 107 months)	Low postoperative HDL-C: shorter OS (HR: 1.76, 1.31-2.38) and a shorter DFS (HR: 2.06, 1.55-2.73).

DFS, disease free survival; FU, follow-up; HDL-C, high density lipoprotein cholesterol; HR, hazard ratio; OS, overall survival

Supplementary Figure S1. Kaplan-Meier survival curves according to HDL-C (A) NHISS cohort. (B) KNUH cohort.

HDL5 means high density lipoprotein cholesterol (HDL-C); 1(<40 mg/dL), 2 (40-49 mg/dL), 3 (50-59 mg/dL), 4 (60-69 mg/dL), and 5 (≥ 70 mg/dL).

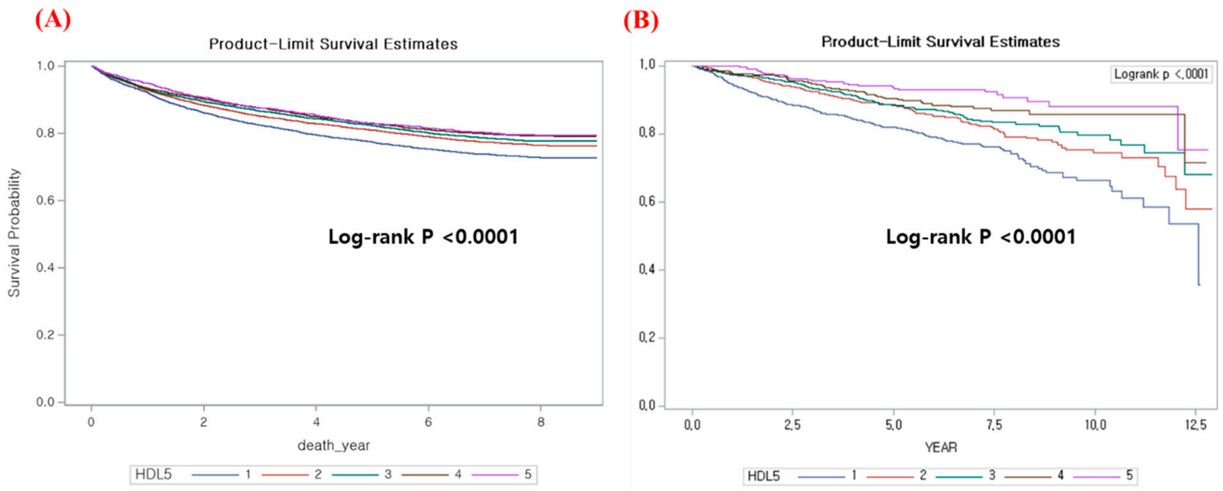

Supplementary Figure S2. Simplified directed acyclic graph (DAG) showing assumed causal structure in our adjusted models.

Directed acyclic graph were constructed at website (http://www.dagitty.net/dags.html#).

Chronic disease means hypertension, diabetes, cerebrovascular disease, heart disease.

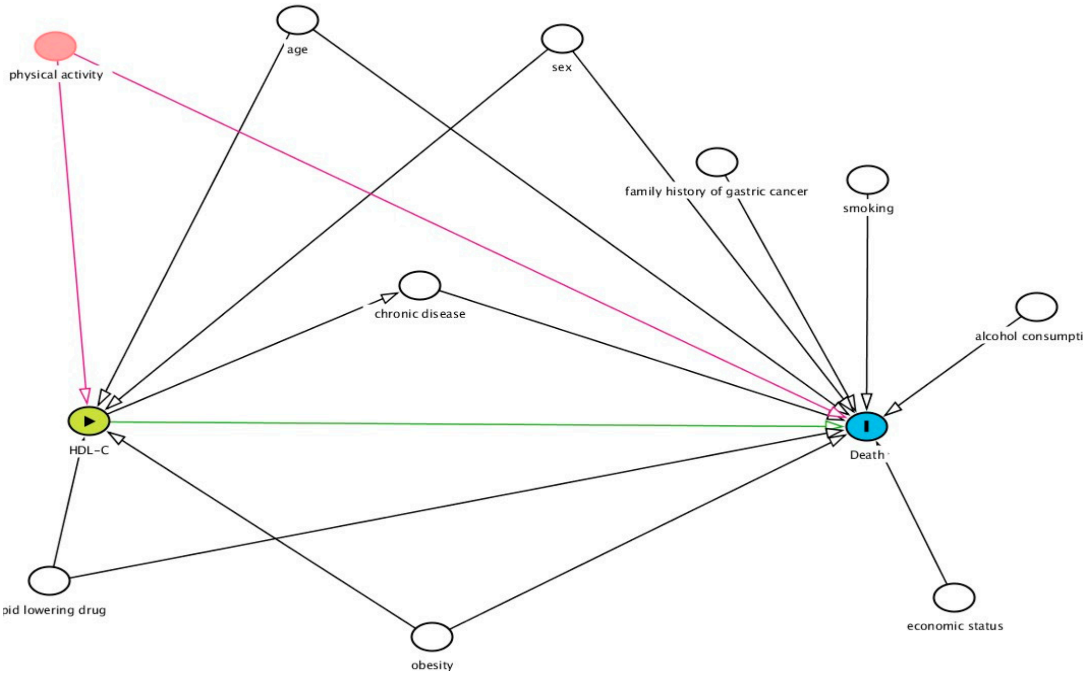

physical activity
age
sex
family history of gastric cancer
smoking
chronic disease
alcohol consumpti
HDL-C
Death
pid lowering drug
economic status
obesity
